# A systematic review on AI/ML approaches against COVID-19 outbreak

**DOI:** 10.1007/s40747-021-00424-8

**Published:** 2021-07-05

**Authors:** Onur Dogan, Sanju Tiwari, M. A. Jabbar, Shankru Guggari

**Affiliations:** 1 Department of Industrial Engineering, Izmir Bakircay University, 35665 Izmir, Turkey; 2 Research Center for Data Analytics and Spatial Data Modeling (RC-DAS), Izmir Bakircay University, 35665 Izmir, Turkey; 3 Department of Computer Science, Universidad Autonoma de Tamaulipas, Ciudad Victoria, Mexico; 4 Vardhaman College of Engineering, Kacharam, India; 5 BMS College of Engineering, Bengaluru, Karnataka India

**Keywords:** COVID-19, Pandemic, Artificial intelligence, Machine learning, Systematic review, Research analysis

## Abstract

A pandemic disease, COVID-19, has caused trouble worldwide by infecting millions of people. The studies that apply artificial intelligence (AI) and machine learning (ML) methods for various purposes against the COVID-19 outbreak have increased because of their significant advantages. Although AI/ML applications provide satisfactory solutions to COVID-19 disease, these solutions can have a wide diversity. This increase in the number of AI/ML studies and diversity in solutions can confuse deciding which AI/ML technique is suitable for which COVID-19 purposes. Because there is no comprehensive review study, this study systematically analyzes and summarizes related studies. A research methodology has been proposed to conduct the systematic literature review for framing the research questions, searching criteria and relevant data extraction. Finally, 264 studies were taken into account after following inclusion and exclusion criteria. This research can be regarded as a key element for epidemic and transmission prediction, diagnosis and detection, and drug/vaccine development. Six research questions are explored with 50 AI/ML approaches in COVID-19, 8 AI/ML methods for patient outcome prediction, 14 AI/ML techniques in disease predictions, along with five AI/ML methods for risk assessment of COVID-19. It also covers AI/ML method in drug development, vaccines for COVID-19, models in COVID-19, datasets and their usage and dataset applications with AI/ML.

## Introduction

COVID-19, novel coronavirus, was announced in Wuhan, China, in December 2019 as a group of fatal respiratory infections and spread quickly as a pandemic [1]. Coronaviruses are pronounced zoonotic in nature and readily spread amongst people [2]. It is still a burning issue to investigate how it is transferred into animal reserves and others [3]. Because no vaccine and decided medication for COVID-19 found until the beginning of 2021, social distancing was stated as the most effective tactic to control and prevent [4]. In addition to social distancing, quarantine is also a critical part of controlling and avoiding the spread of the virus. According to John Hopkins University, the total confirmed cases is 107.5 million, and global death is over 2.3 million in the world [5]. The most affected ten countries are the United States, Brazil, India, Russia, France, Spain, Italy, Turkey, Germany and Colombia. The COVID-19 pandemic is not only a medical contagious but also an economical contagious [6]. Consequently, it is necessary to build an artificial intelligence-based healthcare system because it can quickly and precisely detect cases and avoid the pandemic.

Artificial intelligence (AI) and machine learning (ML) [7] have been recognized as the most potent and hopeful analytical tools in the healthcare domain [8]. Although many health problems are handled by bioinformaticians and statisticians instead of data scientists, a massive amount of data generated in the healthcare creates a necessity to produce more beneficial tools to distinguish exceptional cases from big data. AI computing performs various cognitive functions like humans in a machine to act or react to input data. On the other hand, classical computing has no autonomous intelligence since it requires a hand-code to react to data [9]. It cannot react when an unpredicted state has occurred. Therefore, AI tools continually adapt their reaction to adjust creating their behaviors. In an AI method, computers are designed to analyze, interpret and solve a problem. In machine learning, one of the principal forms of AI, machines learn reactions to use in the future for the same inputs when they face a particular result.

The applicability of AI/ML for epidemiological research of COVID-19 is explored in the literature. Initially, it identifies the relevant key explanatory variables then uses the dimensionality reduction technique to remove redundant features or information. It utilizes Random forest and gradient boosted machine learning models to measure the relative influence of the explanatory variables. This method also determines the interconnections among key explanatory variables, COVID-19 case and death counts. The study shows that air pollution has a high impact on COVID-19 casualties [10]. COVIDetectioNet [11] is proposed to detect the COVID-19. It uses in-depth features generated from the convolution and fully connected layers of the AlexNet architecture. This method has three steps such as pre-learned in-depth features ensemble, feature selection, and classification. It uses the relief algorithm for feature selection and the support vector machine model for classification. This method uses a tenfold cross-validation method to calculate the accuracy.

Deep learning (DL) models are very effective for time-series datasets. In the literature, the prediction of COVID-19 cases using time series data is discussed with DL techniques. Some models, such as long short-term memory (LSTM), are used to predict the time-series datasets. Integration of a convolutional neural network (CNN) and Long short-term memory (LSTM) detects COVID-19 automatically using X-ray images. CNN is used for deep feature extraction, and detection is performed using LSTM using the extracted features [12]. The sample size is a significant challenge with the existing method. Samples contain multiple disease symptoms is one more challenge of this method. Similarly, the prediction of confirmed cases, deaths and recoveries in 10 major countries affected due to COVID-19 is studied. Autoregressive integrated moving average (ARIMA), Support Vector Machine (SVM), LSTM and bidirectional LSTM can be applied for prediction purposes [13]. The superiority of the models can be measured various performance metrics such as root mean square error, mean absolute error and $R^2$ score. Multiple CNN models like ResNet, Inception net V3, Xception net can be used to detect COVID-19 using chest X-ray scans. The small sample size is the main disadvantage of these methods. Due to overfitting, these methods are unable to produce high accuracy [14].

AI/ML techniques have been widely applied to detect new molecules on the way to ascertain COVID-19. Many data scientists adopt AI tools to discover new medicines for the cure, to use X-rays and computational tomography (CT) scans by image processing, to identify the infectious people [15]. AI tools can also develop tracking software to classify people who breach the quarantine rule. AI-embedded thermal cameras and smartphones are practiced to catch infected patients [16]. In a general manner, AI is utilized to identify, track and predict outbreaks by diagnosing the virus. The drones and robots are used to transport food and medicine to related areas or people [17]. Some researches benefit from AI advantages to develop drugs and prepare vaccines [18, 19].

Chest X-ray images have demonstrated a highly effective screening technique for diagnosing the COVID-19. Various hybrid techniques are adopted to detect the COVID-19. Recently, a hybrid DL called COVID-CheXNet is demonstrated to identify the COVID-19. In the beginning, the contrast X-ray image is enhanced using contrast-limited adaptive histogram equalization, and the noise level is reduced with the help of the Butterworth bandpass filter. It uses two pre-trained models such as ResNet34 and HRNet, to identify the COVID-19. Each model’s score is fused to obtain the final class whether the individual is affected by the COVID-19 or not [20]. Similarly, a transfer learning-based hybrid 2D/3D CNN architecture for COVID-19 detection. It uses a pre-trained VGG16 deep model, a shallow 3D CNN. It is also combined with a depth-wise separable convolution layer (to preserve the valuable features) and a spatial pyramid pooling module (to extract multi-level representations). It uses the dataset with three classes such as COVID-19, pneumonia and normal. It achieves reasonable performance concerning sensitivity, specificity and accuracy [21]. A comprehensive study is performed to understand the automatic detection of COVID-19 based on X-ray images using both machine learning and deep learning models. The method’s novelty is demonstrated using COVID-19 vs. Normal dataset and adopt transfer learning to showcase the accuracy. Experimental results indicate that the ResNet50 model performs better as compared to other pre-trained models [22].

**Fig. 1:**
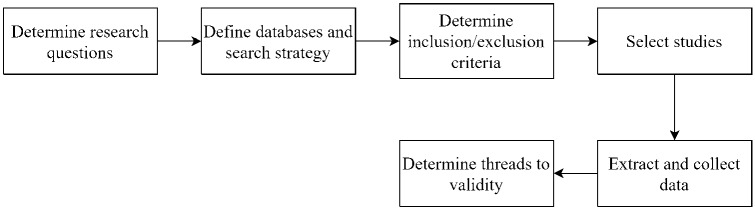
Systematic literature review flowchart

The number of studies on COVID-19 increases day by day because of its popularity and necessity. Researchers need to get a piece of quick information about related studies in this area. In the field of healthcare, AI/ML techniques have been implemented for many applications. For example, because of the availability of MRI, X-ray, and CT images, they have been widely applied for the COVID-19 outbreaks. Although AI/ML applications provide satisfactory solutions to the COVID-19 pandemic, these solutions have a wide diversity in nature. There is no comprehensive study discussing the AI/ML techniques used for the COVID-19 pandemic from different perspectives. Therefore, to fill this scientific gap in the literature, the study’s motivation is to analyze the potential studies using the AI/ML methods [23, 24] for several purposes about the current COVID-19. The study analyzes research on COVID-19 using AI/ML techniques from various perspectives, such as data types, software/tools, applied methods, drug and vaccines. This research’s novelty includes systematically addressing AI/ML techniques as an emerging discipline with tremendous applications in the pandemic. These techniques can be used to understand the nature of this virus and further predict the upcoming issues related to pandemics. This study discusses the significance of AI/ML in resolving the COVID-19 pandemic crisis by examining 264 latest references from seven accessible databases in a systematic way.

Contributions of this study include

- This study mainly focuses on different AI/ML techniques that were applied for the COVID-19 outbreak.
- This study highlights the reasons for applying AI/ML techniques to the pandemic.
- This study explains the data perspective of COVID-19 studies regarding measurement types of study success and data types.
- This review research gives direction to researchers about the various repositories available for COVID-19 outbreak so that researchers can easily access.
- This study focuses on the current situation of drug and vaccine discovery and how AI/ML methods can help in the drug development.
- This study lists various software platforms available to implement AI/ML methods in the COVID-19 outbreak.

This study is structured as follows. The next section gives the research methodology based on seven significant considerations. Research questions, which are critical aspects of the review, are determined. Databases and search strategy are explained together with inclusion and exclusion criteria to select relevant studies. Then data extraction and collections steps are considered. Factors that affect validity to know the strengths and weaknesses of the systematic review are discussed. The subsequent section presents the results and discussions considering defined research questions. Then the limitations of the review are given. Finally, the study is concluded.

## Research methodology

According to Brereton et al. [25], a systematic review of the literature is a method of identifying, evaluating, and interpreting all existing work on a particular research question, subject area or interest. A systematic literature search is conducted with a set of research questions. It aims to answer these questions using a secure, rigorous and auditable methodology [26]. The steps taken in this study are shown in Fig. 1. The process steps in this study are described in the following subsections

### Research questions

The main objective of this systematic literature review is to describe, analyze and synthesize the studies related to the AI/ML implementations in the COVID-19 outbreak. To obtain a more detailed and comprehensive view of the subject, the overall objective is based on the following six research questions (RQs) with motivations.

- RQ 1: What are the most frequently applied AI/ML techniques in COVID-19?
- RQ 2: Why AI/ML approaches are applied in COVID-19?
- RQ 3: What is the data perspective of studies?
- RQ 4: What is the current situation in drugs preparation?
- RQ 5: What software platforms are used?
- RQ 6: Which data sources can be reached?

### Databases and search strategy

Seven online academic search engines were used to find related studies.

- ACM Digital Library
- ArXiv.org
- Elsevier
- IEEE Xplore Digital Library
- PubMed
- Springer
- Wiley Online Library

The search string used to facilitate searching in selected libraries have four dimensions with their sub-domains: AI/ML, study objective, COVID-19, and healthcare.

### Inclusion/exclusion criteria

After collecting the studies, duplicate articles were removed. If there are more than one studies, only the most complete version was chosen. Later, studies were selected using the following inclusion and exclusion criteria to find answers to identified research questions and identify the most appropriate studies.

Inclusion criteria:

- Studies applying at least one AI/ML algorithm
- Studies producing solution to at least one of the COVID-19 problem
- Studies containing experimental work using COVID-19 datasets
- Studies that explicitly address the COVID-19 issue
- Studies written in English only

Exclusion criteria:

- Studies published before 2019
- Extended abstracts and poster work
- Studies that mention AI/ML techniques but are not part of the COVID-19 outbreak
- Studies that mention COVID-19 techniques but do not use AI/ML techniques
- Theoretical studies without application

### Study selection

The articles defined by the search terms from the databases were initially considered only metadata (title and summary). All works related to the subject were scanned. However, since the number of studies found was too large, a second selection was made according to the keywords. The keyword is a way of reducing the time needed to develop the classification scheme and to ensure that the plan considers current work [27]. The full text was examined for the suitability of the articles at the end of the second stage. In the third step, reference lists of related articles were scanned to find extra articles. At the end of the final phase, 264 studies were found eligible for the review.

### Data extraction

A data extraction form was used to collect relevant data from the selected studies to answer research questions. Selected studies were evaluated three times in different days by different authors.

- RQ 1: AI/ML algorithms and techniques used for COVID-19 should be defined.
- RQ 2: Objective of AI/ML approaches should be given.
- RQ 3: The data type, data size, study reliability should be investigated.
- RQ 4: AI architecture for protein structure and drug analysis should be identified.
- RQ 5: AI/ML-based software specific to COVID-19 outbreak should be given.
- RQ 6: Data sources should be searched with a direct link.

**Fig. 2:**
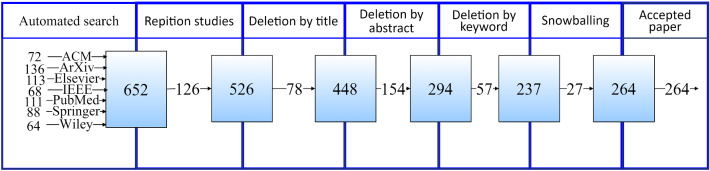
Result of the study selection process

### Data collection

The electronic databases include international indexed journals and conferences searched and defined concerning AI/ML approaches against COVID-19. ACM ($n = 72$), arXiv ($n = 136$), Elsevier ($n=113$), IEEE Xplore ($n=68$), PubMed ($n = 111$), Springer ($n=88$) and Wiley ($n = 64$) databases were scanned. 27 additional studies have been identified by manually searching the reference lists from important studies.

### Threads to validity

It is essential to consider the factors that affect validity to know the strengths and weaknesses of a systematic review [28]. The factors are mainly related to study selection, data extraction and researcher bias in this research.

To find out related studies, the seven search engines mentioned above were scanned. However, it may not be possible to have other relevant works on the results. For this threat, reference lists of selected studies were searched manually to find other related studies, and 27 research were added to the list.

Data extraction is one of the most critical tasks in this work. To reduce the likelihood of extracting wrong data, studies were evaluated twice on different days, and the data needed to answer the RQs were collected.

When selecting and extracting data, it is possible to mention researcher bias [29]. It is a useful systematic review method that one researcher selects studies, and another researcher checks them [30]. The studies in this study were evaluated independently by two researchers and tried to prevent the researcher bias.

## Results and discussion

Relevant studies were determined by applying the research strategy and inclusion/exclusion criteria. For the search on the seven electronic databases described above, 652 candidate studies were selected, as shown in Fig. 2. After removing the first three exclusion criteria and the duplicated studies, 526 articles remained. Then a search based on meta-data (title, keywords and abstract) was done. 237 studies were left after unsuitable studies were eliminated according to the title, abstract and keywords. All of the studies were examined in full text. Since no inconvenience was observed, no elimination was done. As a result, 237 studies related to AI/ML implementations against COVID-19 were agreed suitable for examination. After reviewing these studies’ full text, 27 other studies related to the research were added to the sources through reference lists. Thus, 264 articles were selected directly related to the research.

In recent years, AI has been widely used in various fields of medicine and healthcare [31–33]. Since the outbreak of COVID-19, researchers were successfully used advanced AI technologies in the COVID-19 battle and were achieved significant progress [34–36]. In this survey, a comprehensive review of the contributions of AI/ML in combating COVID-19 is presented. The main scope of AI/ML in COVID-19 research includes the aspects of epidemic and transmission prediction, diagnosis and detection, drug/vaccine development [37].

### RQ 1: What are the most frequently applied AI/ML techniques in COVID-19?

**Fig. 3:**
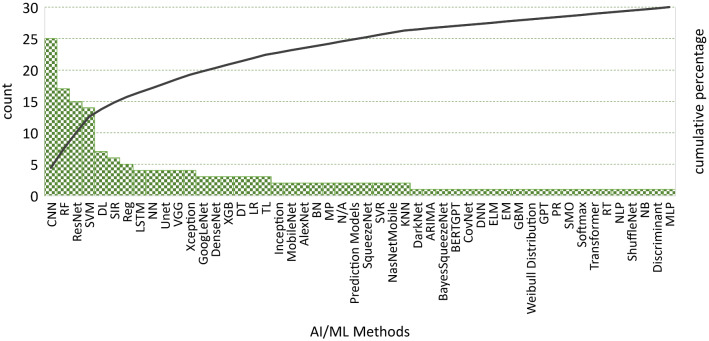
AI/ML approaches in COVID-19

The comparative survey presented in Fig. 3 showed that the convolutional neural network (CNN) model is widely used for medical imaging [38–45]. CNNs are specialized types of neural networks and can be applied to many kinds of data with different dimensions. CNN includes three kinds of layers: convolutional, pooling, and fully connected layers. Convolutional layers constitute the main building blocks of a CNN and summarize the features in an image [46]. CNNs are sensitive to the spatial coherence or local pixel correlations in images. Most of the papers presented in this survey adopted the CNN model because of its high accuracy [47–51]. The results prove that the CNN and deep learning (DL) methods perform best among all the models used in COVID-19 [52, 54–57]. Moreover, CNN was applied together with other methods in many studies such as Unet [58, 59], AlexNet [60] and long short-term memory (LSTM) [61, 62]. ResNet is a pre-trained DL approach that applied more than others [53, 63–67]. However, there some challenges are using CNNs in medical tasks. It is difficult to collect medical images in good quality and sufficient numbers. The availability of labeled data is limited. Collecting and labeling data is a time-consuming process; besides, correctly labeling is critical and depends on specialist experience [68–71]. Random forest (RF) classifier is an ML classifier used by more than 50% of the studies because of its ability to choose the best features for classification [72–78]. SVM is another ML method mostly applied in all scenarios like classification [79–81], prediction [82–84], and diagnosis [85]. Some studies applied more than one pre-trained models and compared their results to find the best method against image recognition [86–89]. Pre-trained networks are composed of two parts. The first part includes a series of convolution and pooling layers, and these layers end with a densely connected classifier. Convolutional feature maps take into consideration of object locations in an input image. On the other hand, densely connected layers at the top of the convolutional base are mostly useless for object detection problems. A pre-trained network is trained on a large dataset, generally on large-scale image classification problems using ResNet, UNet, VGG, Xception, GoogLeNet and XGBoost.

Researchers frequently combined AI/ML techniques and advanced statistical methods to increase the effectiveness of the study outcomes [74, 77, 78, 86, 87, 90–94]. Various ML techniques supported many of the COVID-19 studies [72, 95–100]. For example, Mei et al. [76] developed a joint model that uses CNN and ML (SVM and RF) as a classifier. Susceptible–infectious–recovered (SIR) model and its derivatives such as susceptible–infectious–recovered–deceased (SIRD) or susceptible–exposed–infectious–recovered (SEIR) produces acceptable results using case data [101–104]. Some studies proposed intelligent methodologies including some ML techniques to present effective solutions. For example, Mohammed et al. [37] have evaluated and compared by an intelligent methodology of COVID-19 diagnosis models. They have presented a decision matrix that combined a mix of ten evaluation criteria and twelve diagnostic models for COVID-19. The multi-criteria decision-making method is applied to evaluate and benchmark the various diagnostic models for COVID-19. They have selected SVM classifier as the best diagnosis model for COVID-19.

### RQ 2: Why AI/ML approaches are applied in COVID-19?

**Fig. 4:**
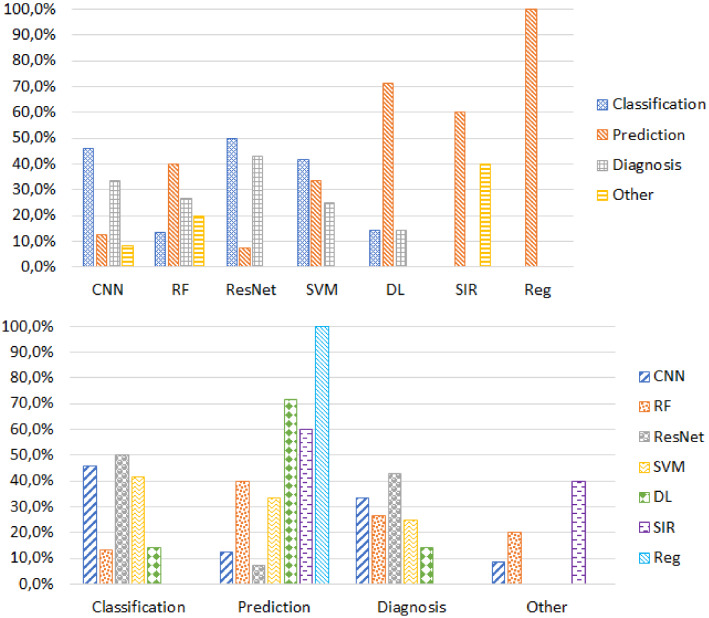
Objectives of AI/ML approaches in COVID-19

AI/ML techniques were used in the COVID-19 pandemic for (1) classification, (2) prediction, (3) diagnosis and (4) other applications like early warnings and alerts. Classification is the most popular aim for applying AI/ML methods [38, 48, 56, 65, 89, 105]. Review results presented in Fig. 4 indicates that most of the models (almost 50% of studies) used ResNet for classification. Recent advancements in DL led to the potential usage of various CNN architectures. Next to ResNet, some authors attempted the CNN model for classification (45% of studies). Few authors also tried to use traditional ML algorithms like SVM and RF for classification of COVID-19 data.

Prediction is the second popular objective in AI/ML approaches [106–111]. Regression analysis is a widely accepted model for prediction purposes (100% of studies) [112]. DL models are another popular prediction approach, which was adopted by 70% of studies. One of the most used mathematical models for the COVID-19 pandemic is SIR frameworks. More than 60% of the studies used the SIR framework for prediction [32, 91, 101–104]. Diagnosis is the third popular AI/ML usage purpose [113–117]. RF and SVM techniques were applied for diagnosis of COVID-19 with nearly 25–30% rates, respectively. As DL-based methods, CNN and ResNet, were used to classify, predict, and diagnose purposes. The results produced by this comprehensive review prove that AI methods are a promising mechanism to use for the current scenario of the COVID-19 pandemic.

Other reasons that concluded from the selected studies to apply AI/ML approaches in COVID-19 are given below.

*Patient outcome prediction* AI tools were developed to predict risk status of contracting the coronavirus. It is critical to know the factors that will put the patients at risk. LSTM is a popular method to predict patient outcome. For example, Obaid et al. [62] proposed a prediction mechanism that uses LSTM to carry this model out on a coronavirus dataset that identified from the records of infections, recovery cases and deaths across the world. Researchers came up with a different proposal to identify the risk factors that will help the clinicians. Some studies proposed models to assess the patients’ severity using the RF and regression model (Reg) [118–120]. Time-series prediction is an important task to predict pandemic diseases. In [121], the authors developed a time series forecasting model using a hybrid machine learning model. Beetle antennae search swarm intelligence algorithm is used for optimization. The proposed model was evaluated using real-time patient data obtained from China by World Health Organization (WHO). The proposed model obtained an $R^2$ score of 0.9763. Table 1 summarizes AI/ML methods for patient outcome prediction.

**Table 1:** AI/ML methods for prediction of patient outcome

Study	Objective	AI/ML approach
[97]	Identify the monocyte ratio and blood pressure in human body	RF
[118]	Predicting hospitalization	RF and Reg
[119]	Severity assessment	RF and Reg
[120]	Severity assessment	Reg
[122]	Identify the high-risk and low-risk patients	Reg
[123]	Identify the mortality risk,	XGBoost
[124]	Patient risk stratification	CNN
[125]	Confirmation of covı cases	LSTM

*XGBoost* extreme gradient boosting

AI and ML models are potentially strong to fight with different pandemic (flu, dengue, zika, cholera, ebola, H1N1, influenza, swine fever) with different methods like classification, forecasting, prediction and pattern recognition. AI/ML tools covering these methods to play an essential role in fighting with the deadly disease [126]. Table 2 shows different AI/ML techniques in disease predictions.

**Table 2:** AI/ML techniques in disease predictions

Study	Disease	AI/ML method	Country
[127]	Dengue fever	CTree	Bangladesh
[128]	Oyster norovirus	GP	USA
[129]	Dengue fever	Reg, NB	India
[130]	H1N1 Flu	NN	Japan
[131]	Influenza	RF	Iran
[132]	Dengue fever	NN	Japan
[133]	Swine Fever	RF	China
[134]	Asthma exacerbations	NB, SVM	USA
[135]	Dementia prediction	SVM	Italy
[136]	Diabetes classification	Reg, NN, NB, KNN, RF	Brazil
[137–139]	Hepatic fibrosis	NB, RF, KNN, SVM, NN	N/A
[140]	Course of depression	Reg	N/A

*CTree* classification tree, *GP* genetic programming, *KNN* K-nearest neighbors, *NB* Naive Bayes, *NN* neural network

*Risk assessment of pandemic* AI/ML models help to assess the risk of the pandemic. DL-based models were developed to predict the duration of the disease [141, 142], community-level risk assessment [143] and transmission prediction [144]. Early risk assessment of COVID-19 patients helps to reduce mortality. Several ML algorithms were developed in the literature. For example, Heldt et al. [145] proposed a model that extracts the informative clinical features from the data. XGBoost algorithm with 100 trees was trained on the dataset. The proposed model obtained (AUC-ROC) scores from 0.76 to 0.87. Table 3 gives an overview of risk assessment of COVID-19 with AI/ML methods.

**Table 3:** AI/ML methods for risk assessment of COVID-19

Study	Objective	AI/ML technique
[141]	Predict the duration of the disease	LSTM
[142]	Transmission prediction	LSTM, RNN
[143]	Community-level risk assessment	GAN
[144]	Transmission prediction	TL
[146]	Disease monitoring	CNN

*GAN* generative adversarial network, *RNN* recurrent neural networks, *TL* transfer learning

**Table 4:** Measurement types of study success

Measurement	Percentage	Min (%)	Max (%)	Measurement	Percentage	Min	Max (%)
Accuracy	31	50	100	Precision	6	79%	99.29
AUC	12	85	99.6	R squared	3	98%	99.7
Explained variance	2	99	99.7	RMSE	1	136.547	
				Sensitivity	20	0.01%	99.62
F1-score	7	79	98.46	Specificity	18	70.7%	99.99

*AUC* area under the curve, *RMSE* root mean square error

*Workload reduction of health professionals* Because the sudden spike of COVID-19-affected patients, healthcare workers have a growing burden. Various AI/ML techniques were proposed for early diagnosis of the disease [147–149]. AI can tackle future challenges and address to reduce the workload of healthcare professionals [150].

*Social control* With high transmissibility of COVID-19, many countries adopted AI for pandemic management [151] and are successful in reducing the mortality rate. For example, a predictive model for mortality rate in COVID-19 using ML was developed by Booth et al [152]. Model identified the prognostic serum biomarkers in COVID-19 patients. Five serum parameters were used in the data set using a support vector classifier for classification. The proposed model achieved 91% specificity and 91% sensitivity. AI can facilitate the management of contact tracing, quarantine and self-isolation of people, screening for infection [153, 154]. AI-based drones were used to enforce social isolation [155].

*Early warnings and alerts* AI is a potential tool to fight against COVID-19, and AI-based systems are used in spotting COVID-19 disease outbreaks. Bots based on AI were used to predict the possible outbreak [156, 157]. Before the WHO (World Health Organization) sounded an alarm on the possible outbreak of COVID-19, an AI bot named “BlueDOT” [158] alerted employees’ possible outbreak of a pandemic. A similar bot, called “Health Map”, developed in the USA sounded the alarm for possible outbreak [159].

### RQ 3: What is the data perspective of the research?

Table 4 gives the validity measurement types of researches. Most of the studies validated the research results by accuracy [77, 160–163]. Accuracy scores vary from 50 to 100%. However, these results are not the final output of these studies. For example, Elgendi et al. [86] and Hemdan et al. [87] applied various pre-trained AI methods. Whereas Elgendi et al. [86] reached 100% accuracy rates using ResNet-50, DarkNet-53, VGG-19, DenseNet-201, ResNet-18, ResNet-101, and GoogLeNet, Hemdan et al. [87] obtained a 50% accuracy score by InceptionV3. 82% of the research were tested the validity by three measurement types: accuracy, precision and sensitivity [58, 164].

**Table 5:** Data types used in the COVID studies

Data type	Percentage	Min	Max
CT	49	106 images	16,756 images
X-ray	35	50 images	15,085 images
Case data	16	14 days	77 days

**Table 6:** AI/ML method in drug development

Study	Drug type	AI method	AI/ML objective	Potential drugs
[178]	SARS-CoV-2 inhibitors	ChemAI	Predict inhibitory effects of molecules	30,000 top-ranked compounds
[179]	Antiviral drugs	MT-DTI	Predict commercially available antiviral drugs	Atazanavir, Remdesivir, and Efavirenz
[180]	Antiviral drugs	MT-DTI	Predict binding affinity between drugs and protein target	Remdesivir, Atazanavir, Efavirenz, Ritonavir, Dolutegravir, Kaletra
[181]	Anti-COVID-19 drugs	CNN, LSTM, MLP	Generate SMILES strings and molecules	110 drugs
[182]	Targeted proteins of SARS-CoV-2	DL	Predict binding between drugs and protein	10 drugs
[183]	SARS-CoV-2 drug	NN, NB	Construct drug likelihood prediction model	3 drugs
[184]	2019-nCoV	DL	Generate new molecular structures for 3CLpro$^\mathrm{{a}}$ structures	100 molecules

$^\mathrm{{a}}$The viral main proteinase of coronavirus

Table 5 represents data types and their statistics. Almost half of the COVID-19 works that benefit from AI/ML techniques analyzed CT images [59, 165–170]. X-ray is the second popular data type with a rate 35% [31, 66, 89, 162, 171–174]. A massive data size scale was used in those studies, ranging from 106 to 16,756 CT images and 50–15,085 X-ray images. Some studies focused on case data such as death and recovery numbers between a specific period [77, 90, 91, 175, 176]. Other data types such as dialogue data [92, 177], genome data [99], symptoms [72], blood data [74, 98] were excluded in Table 5 because they were measured below 5% of the studies.

### RQ 4: What is the current situation in drug preparation?

Due to the rapidly spreading across to the world and the lack of effective treatment options, drug developers have adopted the various strategies to fast track the drug discovery. Whereas some studies applied AI/ML techniques to predict, some of them analyzed the molecular structure of coronavirus because drug discovery is an expansive and lengthy process. Table 6 represents the drug studies against to COVID-19.

AI is a cost-effective and fast tool in drug discovery to fight against COVID-19. Shin et al. [180] proposed a Molecule Transformer Drug Target Interaction (MT-DTI) model that provides low-cost drugs and personalized medicines with multi-layered protein. MT-DTI was also applied to predict commercially available drugs [179]. This is the drug-target interaction model that uses deep learning. The result showed that Atazanavir, Remdesivir, and Efavirenz are suitable to fight against SARS-CoV-2. Hofmarcher et al. [178] proposed a DL model for drug discovery by predicting the inhibitory effects of molecules. Initially, they identified one billion molecules from the ZINC database for screening and ranking, and further molecules were reduced to 30K.

**Fig. 5:**
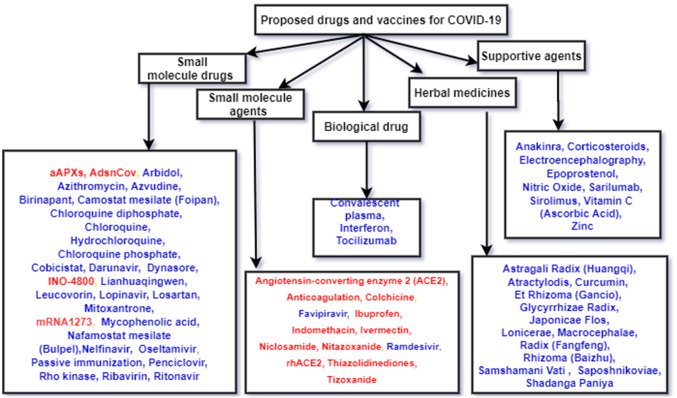
Drugs and vaccines for COVID-19

Some studies identified the drug compounds to fight against SARS CoV-2 coronavirus. Kadioglu et al. [183] identified three potential drugs for COVID-19 by adopting in silico methods to identify novel drugs using an AI model based on NB and NN. Hu et al. [182] identified ten drugs as potential inhibitors fight against SARS-CoV-2 by predicting the binding between drugs and protein using DL methods. Figure 5 summaries some candidate drugs or vaccines to treat this disease, which includes small molecule drugs, small molecule agents, herbal medicines and biological products [185–190]. Blue texts show the drug developments, whereas green texts refer vaccine developments.

Both small molecule drugs and small molecule agents are more potential drugs for COVID-19 [191]. Small molecule drugs like Lopinavir/Ritonavir and Ribavirin were used for the antiretroviral activity. On the other hand, Chloroquine phosphate and Arbidol were used to synthesize viral DNA or RNA. Small molecule agents such as Remdisivir, Favipiravir were used as an RdRp inhibitor. Similarly, biological products were used as a monoclonal antibody (Tocilizumab) or passive immunity boosters (Convalescent plasma). Some studies treated the COVID-19 with the help of a combination of drugs such as (hydroxychloroquine, azithromycin), (azithromycin, nitazoxanide), (favipiravir, hydroxychloroquine) and (favipiravir, azithromycin) [192].

Scientists are looking for a vaccine at least 95% effective to stop the pandemic [193]. AI techniques were widely used in the design of vaccines against SARS-CoV-2 [194, 195]. Some studies utilized AI approaches to obtain protein sequences [196] and nucleotide sequences [197]. Epitope prediction using AI/ML techniques were also popular in vaccine development against COVID-19 [196–201].

### RQ 5: What software platforms are used?

Practitioners encountered severe challenges in the detection of Ncov-2019 because SAR-CoV-2 viruses spread rapidly. Reverse Transcription Polymerase Chain Reaction (RT-PCR) approach is not applicable due to some obstructions [202]. The shortcomings of RT-PCR can be obviated by analyzing medical images because developing digital technologies help prevent diseases by applying statistics, machine learning, and artificial intelligence models [203]. Table 7 presents several models and software platforms. These models’ capability was provided in a broad range of uses; from disease detection and prediction to social control. Applications involve real-time data analysis for disease detection and diagnosis, treatment monitoring, prediction of cases and mortality, and drugs/vaccines development [204]. Except from the studies in the table, some studies used more than one software such as Python and Excel [205], Python and R [118, 206], MATLAB and Excel [207].

**Table 7:** Models in COVID-19 with software platform

Software	Study	Model	Data source
Python	[207]	SIR, SDM, PA	Worldometers
	[208]	Regression model	MoHFW, covid19india.org
	[209]	Pre-trained CNN	GitHub, Kaggle, Open-I repository
	[160]	CT radiomics	GitHub
	[205]	Regression model	covid19india.org, WHO
R	[210]	SIRD and SVM	Worldometers
	[211]	ARIMA, SIR	Johns Hopkins U.
	[212]	Regression model	Worldometers
	[213]	SIR	Johns Hopkins U.
	[214]	Regression model	Worldometer, covid19India.org
	[163]	Hybrid model approach	Worldometers, ourworldindata.org
	[215]	Regression model	MoHFW, John Hopkins U
	[216]	Regression model	WHO, Historical weather
Not Given	[217]	Regression model, MLP	Kaggle
	[218]	ARIMA, SVM	WHO
	[219]	Fractional mathematical model	N/A
	[220]	AP, TB	WHO, Worldometers
	[221]	Exponential growth model	MoHFW, WHO, covid19india.org
	[222]	SIR, Network model	COVID19USA
	[223]	Regression model	John Hopkins U

*AP* arithmetic projection, *ARIMA* autoregressive integrated moving average, *MoHFW* Ministry of Health and Family Welfare, Government of India, *MLP* Multilayer perceptron, *PA* propagation analysis, *SDM* social distancing matrix, *TB* tree-based model

### RQ 6: Which data sources can be reached?

Data are presented as an essential aspect of implementing scientific methods. The research community always follows two approaches: closed source or open source [224]. Closed source is considered for proprietary objects, whereas open source leads to more precious quality, transparency, verifiability, usability [225, 226]. In the COVID-19 pandemic, the open-source approach is considered more effective for mitigating and detecting the virus due to its prior symptoms. It is highlighted that the COVID-19 pandemic needs a collaborative and unified approach along with open-source data, so the scientific community can get transparent and valid research [227, 228]. Different datasets were presented to combat with the COVID-19 pandemic in different ways [224].

Three main types of datasets in COVID-19 were used, textual data, medical data and speech data. Textual data represents dashboard, mobility data, case reports, social media posts and articles. Medical data generally presents diagnosis and screening of COVID-19 patients since medical images consider X-rays, CT scans, ultrasound or MRI (Magnetic Resonance Imaging). Most of the datasets represent CT scans, X-rays, and AI/ML techniques applied to predict resources in the future. Speech datasets help to detect and diagnose by cough sound, breathing rate and stress detection techniques.

Most of the datasets were stored on different repositories, such as Github and Kaggle. Table 8 presents 18 textual datasets, nine medical datasets and seven speech datasets.

**Table 8:** Datasets and their details

Textual data sets		Medical datasets	
Data sets	Explanation	Data sets	Explanation
T1 [229]	Datahub repository	M1 [230]	COVID-19 CT scans of Chinese hospitals with an online repository
T2 [231]	Github repository of the data	M2 [232]	Dataset consists of 20 COVID-19 CT scans
T3 [233]	Medical community	M3 [234]	Segmentation benchmark
T4 [235]	Real-time interactive dashboard	M4 [236]	COVID-19 CT segmentation dataset
T5 [237]	Open source datasets	M5 [238]	Images from a repository
T6 [239]	crowd-sourced list of open access COVID-19 projects	M6 [240]	3D CT scans of confirmed cases
T7 [241]	Country specific case reports and articles	M7 [242]	COVID-19 positive and suspected patients
T8 [243]	Demographic database	M8 [244]	Analyzing radiographical images
T9 [245]	Real-time and historical mobility data from Wuhan	M9 [246]	Repository for COVID-19 radiographic images
T10 [247]	Real-time data	Speech and audio datasets	
T11 [248]	Data sets of Twitter posts	Data sets	Explanation
T12 [249]	Data sets of Twitter posts	S1 [250]	Web application for data collection
T13 [251]	Web search portal for dataset of scholarly articles	S2 [252]	Open source voice dataset
T14 [253]	Google mobility reports	S3 [254]	Collection of the cough data
T15 [255]	Data set available on mobility based on user requests to location services	S4 [256]	Collection of the cough data
T16 [257]	Web application identifying mobility patterns across the U.S	S5 [258]	Collection of the cough data
T17 [259]	Mobility data from Baidu location services	S6 [260]	Data collection for cough data
T18 [261]	Google location services	S7 [262]	Repository for the cough data

Total 18 textual datasets were discussed to show the relevancy of different purposes. These datasets consider COVID-19 case reports, report analysis, mobility data, social media data, scholarly articles, tweets, non-pharmaceutical interventions (NPI). Several studies maintained and shared the epidemiological data of COVID-19 cases in China [225, 263]. COVID-19 case reports include different details like (a) symptoms of the disease, (b) dates of patient admission, date of infection confirmation, travel dates, (c) other information like resources of food [263]. They were presented to analyze the transmission, testing, forecasting and death cases [264–269]. Some studies evaluated and investigated human mobility, travel restriction, social distancing and control measure [270–274]. Social media data and scholarly articles were also collected to present different textual data such as emotions and worries [275–281] and scientific article data from existing studies [282–286]. Tweets also provide collected textual data. Several studies collected twitter datasets to identify the pandemic information from a social aspect and analyze human behavior [278, 279, 287]. NPI is considered as different sets of measures accepted by governments to prevent the COVID-19 pandemic. The NPI effect was analyzed for COVID-19 cases [288]. Mobility datasets are significant to provide the information of infected cases and also helpful to diagnose the response of societies in NPI restrictions. Several open-source datasets provide information with dynamic features.

Medical datasets, which include CT and X-ray images, are essential in diagnosis of COVID-19. Studies based on COVID-19 diagnosis used different datasets for CT-Scan [34, 89, 165, 289–297] and X-ray [20, 78, 87, 298, 299] images by different AI/ML techniques [160, 300, 301]. The study of Sharma and his colleagues [302] distributed the original image dataset into 10% external validation dataset-I and 90% training dataset as Dataset-II. Dataset-I has 35 images, and Dataset-II has 317 images and generated a total of 27 different types of training and validation datasets for chest X-ray images. Out of these datasets, one dataset includes real images, and 26 datasets consist of single augmentation images. All these 27 datasets were used to train and validate the 29 types of chest X-ray classification models. A comprehensive study was performed to understand the performance of automatic detection of COVID-19 based on medical images [22]. This study uses COVID-19 and normal X-ray images and adopts transfer learning to increase the accuracy. To make general framework and avoid overfitting, different training policies are adopted using AdaGrad algorithm. A hybrid deep learning framework COVID-CheXNet has been proposed by Al-Waisy et al. [20] to reduce the load on radiologists and control of the pandemic. This model helps to diagnose the COVID-19 virus in chest X-ray images and is composed of four primary stages: image pre-processing, image classification, features extraction and fusion. Mohammed et al. [22] have proposed an automatic prediction to identify COVID-19 for discriminating automatically between normal and COVID-19 infected people in X-ray images. To accomplish this, they used traditional ML methods such as SVM, NN, DT and kNN techniques. They also applied deep learning models such as ResNet50, MobileNets V2, DarkNet, GoogleNet, and Xception.

Speech or audio datasets help to detect and diagnosis of infection by three different method such as cough sound analysis [303–305], breathing rate analysis [306–309] and stress detection [310–312]. Cough sounds can identify a COVID-19 infected case by applying ML techniques. Breathing rate can be identified by speech, resulting in COVID-19 patient screening. Stress detection also helps to identify the cases that person suffer from mental health issues and symptoms of COVID-19. These methods can be done by remote medical care or smart devices. AI/ML techniques are successfully applied for extracting features and classify new inputs based on model training.

**Table 9:** Dataset applications with AI/ML

Study	Application	Methods	Database
[332]	COVID-19 diagnosis	DenseNet, TL	Medical
[290]	COVID-19 diagnosis	Deep CNN	Medical
[87]	COVID-19 diagnosis	Deep learning	Medical
[78]	COVID-19 diagnosis	CNN, TL	Medical
[31]	COVID-19 diagnosis	CNN	Medical
[301]	COVID-19 diagnosis	CNN	Medical
[271]	Cases exported from China	Statistical	Medical
[266]	Correcting under reported cases	Statistical	Textual
[273]	International travel control analysis	Statistical	Textual
[274]	COVID-19 transmission control	Regression analysis	Textual
[333]	Community transmission	Expectation maximization	Textual
[334]	Community transmission	Bayesian approach	Textual
[276]	Social dynamics data	Statistical analysis	Textual
[335]	Perception and policies	Proposed NLP	Textual
[281]	COVID-19 symptom identification	Data mining	Textual
[304]	COVID-19 diagnosis	Boosting Trees, SVM	Speech
[305]	COVID-19 diagnosis	N/A	Speech
[309]	COVID-19 speech analysis	SVM with linear kernel	Speech
[279]	Government and Media Tweets	N/A	Textual
[277]	Conversation dynamics	N/A	Textual

Table 9 gives a tabular and descriptive survey for various open source datasets. This table covers 20 datasets with different data-types such as X-ray, CT Scans, Ultra-sound, case data, tweets, voice data. These datasets were applied different methods with different applications. For example, CNN, SVM and TL were applied for diagnosis [38, 165, 313–315]. Bayesian approach method was applied in community transmission [316–321], while data mining methods [322–327] were used for symptoms identifications. Regression analysis methods [148, 328–331] were used for transmission control analysis.

## Limitations

Some limitations of the current research should be accepted. The research is limited to selected search terms, databases and selection criteria.

This research was conducted in a certain period of time. However, the number of studies on COVID-19 increases day by day because of its popularity and necessity. Because a systematic literature review was presented with this research, it is necessary to limit the research content. To decrease the effect of this situation, the inclusion and exclusion questions were prepared to select the studies published in the research period.

Seven online databases were scanned for the review. However, other databases can be scanned. If the research is to be expanded, the number of databases can be increased.

Apart from selected studies in this research, there are many different studies. It should not be forgotten that some criteria were set for narrowing the research scope. For example, studies that do not mention the algorithm applied in the implementation or do not give details were ignored. Applied AI/ML studies are generally implemented for different purposes without considering COVID-19 problems. Therefore, COVID-19 problems are not explicitly stated in the publications. By evaluating each study individually, it was determined which problem discussed. At this stage, there may be unobserved publications.

**Table 10:** Abbreviations used in this study

Abbr.	Explanation	Abbr.	Explanation
AI	Artificial intelligence	NN	Neural network
AP	Arithmetic projection	NPI	Non-pharmaceutical interventions
ARIMA	Autoregressive integrated moving average	PA	Propagation analysis
AUC	Area under curve	Reg	Regression models
CNN	Convolutional neural network	RF	Random forest
COVID-19	Coronavirus disease 2019	RMSE	Root mean square error
CT	Computational tomography	RNN	Recurrent neural networks
CTree	Classification tree	RQ	Research questions
DL	Deep learning	RT-PCR	Reverse transcription polymerase chain reaction
GAN	Generative adversarial network	SDM	Social distancing matrix
GP	Genetic programming	SEIR	Susceptible, exposed, infectious, recovered
KNN	K-Nearest Neighbor	SIR	Susceptible, infectious, recovered models
LSTM	Long short-term memory	SIRD	Susceptible, infectious, recovered, deceased
ML	Machine learning	SVM	Support vector machine
MLP	Multilayer perceptron	TB	Tree-based
MRI	Magnetic resonance imaging	TL	Transfer learning
MT-DTI	Molecule transformer drug target interaction	WHO	World Health Organization
NB	Naive Bayes	XGBoost	Extreme gradient boosting

## Conclusion

This systematic review study investigates 264 studies from seven accessible databases to find answers for six significant research questions. This research aims to explore and organize potential literature so that practitioners, academicians, and researchers can easily access the existing methods, applications, and datasets. The main contribution of this research to identify the AI/ML methods and techniques for disease prediction, measurement and data types, AI/ML method in drug development, available drug and vaccines, and existing models and datasets for the COVID-19 pandemic. CNN, RF, ResNet and SVM approaches are the most used AI/ML approaches against COVID-19. These approaches were applied for various purposes. Classification, prediction and diagnosis are the most popular AI/ML objectives. ResNet applied for classification and diagnosis, whereas regression is used for prediction studies. Apart from these objectives, previous studies benefited from the advantages of AI/ML tools for several additional purposes, such as patient outcome prediction, risk assessment, workload reduction of health professionals, social control and early warnings and alerts. This study concludes that the methods’ success varies widely. Nine major measurement types were considered to evaluate models’ success. Accuracy, sensitivity and specificity were measured 69% of studies. 84% of studies used either CT or X-ray images between 50 and near to 17,000. Case data are the third popular data type with a rate of 16% up to 77 days. Python and R the most preferred software platform to apply AI/ML methods. Some studies used Matlab, Microsoft Excel and more than one software. Data were stored in three main categories, textual, medical, and speech. Because the research has review borders, it has some limitations that were discussed in the study.

This study is most significant for new practitioners and researchers who plan to develop an AI/ML model or drug for COVID-19. They can reuse existing models and drugs rather than design from scratch and save time for doing potential research and future studies. Besides, this research provides a backbone for different aspects such as disease diagnosis and detection, drug and vaccine development, AI/ML models and techniques. The conducted literature provides comprehensive details of AI’s potential and existing contribution to combating the pandemic.

As it is understood from the literature review, many researchers applied CNN models. The main reason can be that they are powerful for the spatial coherence or local pixel correlations in medical images. CNN technique was usually applied for either classification or diagnosis. However, authors should remind aforementioned drawbacks before applying CNN for COVID-19 studies.

For further research, the authors can focus on several points. First of all, researchers can scan other databases such as ERIC, DOAJ and JSTOR. Some additional research questions can be investigated to clarify interesting and meaningful results.

## Abbreviation

Table 10 presents the abbreviations used in the study.
